# Biological Activities of Natural Products

**DOI:** 10.3390/molecules25235769

**Published:** 2020-12-07

**Authors:** Halina Maria Ekiert, Agnieszka Szopa

**Affiliations:** Chair and Department of Pharmaceutical Botany of Pharmaceutical Faculty, Jagiellonian University Collegium Medicum, 9 Medyczna Street, 30-688 Kraków, Poland

Natural products and their biological activities are currently a subject of great interest in the pharmaceutical, health food, and cosmetics industries, and numbers of scientific studies in this field are increasing rapidly. One reflection of this trend is the fact that *Molecules* has recently published several special issues devoted to various aspects of natural products.

The plant kingdom is a still insufficiently known reservoir of a wealth of bioactive natural compounds. Other organisms, such as microorganisms, fungi, and animals (including insects), can also supply us with valuable biologically active products.

A majority of contemporary studies on the biological activity of natural products focus on various species of plants. Many are devoted to the search for natural compounds, in particular those which demonstrate anticancer, immunostimulating, anti-inflammatory, antioxidant, neuroprotective, and hepatoprotective activities. Others also document newly discovered activities of medicinal plants whose benefits were previously known only fragmentarily.

Numerous papers report on the use of sophisticated research methods to verify the therapeutic applications of “exotic” plant species long known in the traditional medicine of some ethnic groups and/or populations. Some studies investigate the use potential of “new” previously unstudied plant species. A lot of studies attempt to discover and explain the mechanisms of proven biological actions and to answer the question of which compounds are responsible for the documented biological activities of particular extracts.

This Special Issue takes the state of our knowledge one step further, with contributions supplied by scientists from all over the world comprising 27 original papers as well as three review articles.

It is our hope, as the guest editors of this Special Issue that the readers of *Molecules* will enjoy browsing through a wide variety of topics addressed by the authors and to gain greater insight into the main directions of current research in the area of the biological activity of natural products.

A number of the original articles published in this issue look at anti-cancer activity and present the results of in vitro and in vivo studies.

Subhawa et al. [1] documented in vitro the mechanism of the anti-breast carcinoma effect (on 2 lines of human breast cancer cells) of ethanolic extracts from leaves and stems of *Houttuynia cordata* and *Piper ribesiodes*, two East-Asian plant species with already known anticancer activity. 

Rojas-Armas et al. [2] present the results of in vivo studies (on female rats), which demonstrated the antitumor effect of essential oil from the South-Asian grass species *Cymbopogon citratus* and in parallel also of carvacrol in breast cancer induced experimentally. Both the essential oil and carvacrol had an antitumor effect irrespective of the doses. The authors suggest that it may be citral that is responsible for the antitumor effect of the studied essential oil. In an antioxidant assay (DPPH), carvacral exhibited higher activity than essential oil.

Huang et al. [3] evidenced the antitumor effect of fresh bark extract of *Cedrus atlantica*, a forest tree species distributed in northern Africa. Its anticancer activity against human hepatocellular carcinoma (HCC) was confirmed in in vitro and in vivo studies. The lipid fraction from a steam-distilled preparation of the bark, which contains thujopsene and α-cedrene, as the main compounds showed marked dose-dependent inhibitory effects on HCC cells. The extract was less cytotoxic to normal cells (in vitro tests) and induced no obvious pathological changes in the morphology or body weight of the experimental animals (female nude mice), suggesting an absence of toxicity. The authors recommended the investigated extract as a potential anticancer therapeutic agent.

Kowalik et al. [4] documented the influence of extracts from aerial parts of *Potentilla alba*, differing in polarity, on human colon cancer cells and non-cancer colon epithelial cells. This plant species is used in Ukrainian medicine to reduce the blood level of thyroxin. In the present work, the authors revealed the cytotoxic properties of the extracts and their antioxidant activity (DPPH and FRAP tests). Importantly, the extracts showed opposite effects on the proliferation of cancer and normal cells. 

Huang et al. [5] draw attention to the prospects of combination therapy as a new trend in the treatment of oral cancer (a type of head and neck cancer). The authors demonstrated for the first time that crude extract of liquid oil from *Juniperus indica* (a plant species famous in the traditional medicine of Taiwan) exhibited anticancer capacity. It synergizes with cisplatin to treat oral cancer. The authors present the results of in vitro comparative cytotoxicity studies on a human oral cancer cell lines and animal endothelial non-cancer cell line.

One of the articles written by Ilina et al. [6] established the immunomodulating activity of *Galium aparine* (plant species common in Europe, North Africa, and Asia) and partly justified the traditional use of this plant species in skin and wound infections. The studies tested the activity of a raw infusion of the aerial parts of the plant and bioactive fractions (with polysaccharide complex, polyphenolic complex, and pectins). All fractions significantly stimulated the transformation of immunocompetent blood cells. All samples also displayed antioxidant activity (DPPH, NO, H_2_O_2_ assays). 

The anti-inflammatory activity of the extracts and/or isolated compounds was the next area of scientific interest of some authors.

A good example is the article by Kłeczek et al., [7] which deals with chemical analysis of aerial parts of *Xerolekia speciosissima*. The root extracts of this plant species, which is endemic to the pre-Alpine area, were investigated earlier by the authors. In the present work, more than 25 compounds were identified in its hydroalcoholic and chloroform extracts. One compound isolated from chloroform extract, namely 7,10-diisobutyryloxy-8,9-epoxythymyl isobutyrate showed anti-inflammatory activity. 

Anti-inflammatory activity and also antioxidant activity were the focus of the studies performed by Pudziuvelyte et al. [8]. The authors investigated ethanolic extracts of various organs of *Elsholtzia ciliata*, a plant species widely distributed in China, Korea, and Europe. The results documented the anti-inflammatory activity of the examined extracts (in mouse peritoneal macrophage cell culture) and their antioxidant activity (DPPH, ABTS, FRAP, and CUPRAC assays). Additionally, the authors explored for the first time antioxidant efficiency with the use of HPLC-ABTS post-column method, which can be used to identify active compounds, such as avicularin, chlorogenic acid, and rosmarinic acid, in the extracts.

The antioxidant activity was also the subject of studies by Tavarini et al. [9]. The authors focused on the best cultivating conditions for production of *Stevia rebaudiana* (plant species with South-American origin) bioactive compounds. They studied the influence of arbuscular mycorrhizal fungi symbiosis, phosphorus fertilization, and harvest time on the accumulation of secondary metabolites, including stevia glycosides, flavonoids, and total phenols, in the plant leaves. All investigated factors influenced the stevia metabolite yield. The authors documented that in vitro antioxidant capacity (FRAP and ORAC assays) depended on polyphenols and glycosides, while cellular antioxidant activity (CAA) mostly on total phenols. The authors proposed the best cultivation conditions to obtain a high-value plant raw material for the food and cosmetics industries. 

Some articles concentrate on the neuroprotective and hepatoprotective activity of plant-derived substances. Mohamed et al. [10] evidenced the neuroprotective effect of 7-geranyloxycinnamic acid, isolated from *Melicope lunu-ankenda* leaves, against H_2_O_2_-induced neurotoxicity in human neuroblastoma cells. This compound protected the cells against H_2_O_2_-induced apoptosis. It is a good candidate for prevention and treatment of neurodegenerative diseases.

Czernicka et al. [11] looked for acetylcholinesterase inhibitors among *Zingiber officinale* terpenoids. Diethyl-ether extract of fresh ginger rhizomes was shown to contain three bioactive compounds (ar-curcumene, α-sesquiphellandrene, and α-zingiberene). The authors searched for the best extraction technique. Hydrodistillation, supercritical fluid extraction, and shaking maceration yielded high amounts of the volatile components. To identify the potentially active inhibitors, the TLC-bioautography technique coupled with TLC-HPLC-MS approach was used by the authors.

Lai et al. [12] presented a potential protective or restorative action of moscatilin on glycation that could cause various neurodegenerative diseases. Moscatilin could be obtained from orchids of *Dendrobium* species, the famous source of this compound widespread in Southeast Asia. The pathology associated with neurodegeneration involves hyperglycemia accelerating the formation of advanced glycation endproducts (AGEs). The authors presented the results, which indicated that moscatilin could protect human neuroblastoma cells against AGE-induced injury and mitochondrial dysfunction. 

Piotrowska-Kempisty et al. [13] evaluated hepatoprotective effect of *Aronia melanocarpa* juice and sylimarin (positive control) in rats chronically treated with CCl_4_. The anti-fibrosis effect was expected by the authors. After application of aronia juice, hepatic lipid peroxidation was suppressed. Increased activity of hepatic antioxidant enzymes was not translated into an attenuation of liver fibrosis. On the other hand, histological examination revealed the beneficial effect of aronia juice. In the applied experimental model of fibrosis, the effect of silymarin was also very limited.

Several articles are devoted to verification and confirmation of traditional applications of plant species by some ethnic groups and/or populations. 

One of them written by Rudra et al. [14] verifies the antidiarrheal effect of various solvent extracts from *Tetrastigma leucostaphylum* in in vivo studies (mouse). This liana (especially its leaves) is used in ethnomedicine of certain ethnic groups in Bangladesh. All tested extracts and particularly dichloromethane extract showed consistent and dose-dependent antidiarrheal efficacy. All extracts exhibited cytotoxicity in in vitro studies. Computational analysis revealed promising binding affinities of the identified constituents (GC-MS) for various receptors. The drug-like behavior of the identified constituents (10 compounds) was tested through ADME/T analysis.

Elmi et al. [15] concentrated their studies on the chemical composition and biological (antioxidant, antibacterial, and cytotoxic) activity of aqueous and methanolic extracts of the bark of *Acacia seyal*, which is a traditional medicinal plant used by the population of Djibouti in Randa in the form of aqueous extracts as an anti-infectious drug. The bark is rich in tannins, which could be responsible for its documented antioxidant activity (DPPH, ABTS, and FRAP tests). Its sterols and oleamide could be responsible for its antibacterial activity against *Staphylococcus aureus*. The cytotoxicity of aqueous bark extract against fibroblasts indicated that the safety of the extracts used by the Djibouti population should be verified. 

The next work by Imam et al. [16] presents the results concerning the preparation of piperine complexes with better aqueous solubility than piperine alone. The authors managed to prepare stable binary inclusion complex (piperine and hydroxypropyl β-cyclodextrin) and ternary inclusion complex (containing additionally D-α-tocopheryl polyethylene glycol succinate) with elevated solubility and dissolution. The authors proved an enhanced antioxidant and antimicrobial activity of the ternary complex. The results of the authors are supported by molecular docking studies. These results provide a good support for application of the ternary complex as a functional ingredient of drugs and food.

The article by Palla et al. [17] focuses on the antimalarial activity of 35 compounds, synthesized by them as a series of fosmidomycin (FSM) derivatives and conjugates with artemisinin and aminochloroquinoline. The authors evaluated efficacy of the synthesized compounds against the chloroquine-resistant *Plasmodium falciparum*. They managed to synthesize four new compounds with higher antimalarial activity than fosmidomycin. 

A different problem was investigated by Silva et al. [18]. The authors looked for the effective compounds against mosquito *Aedes aegypti*, which is the vector for arboviral diseases (dengue, Zika, yellow fever, and chikungunya). They tested the *Connarus suberosus* (a species common in the Cerrado biome in Brazil) root wood ethyl acetate extract and 28 compounds from the quinone class for development of a larvicide. Embelin and rapanone, isolated from the plant extract, were active against mosquito third-instar larvae. Eight quinones demonstrated larvicidal activity. Tectoquinone was the most active compound and was selected for formulation trials to develop a non-toxic prototype larvicide to control the mosquito development.

The results of two articles are the proposal for cosmetic use.

Jang et al. [19] isolated sesquiterpene (6-*O*-isobutyryl-britannilactone) with a high anti-melanogenesis activity from ethanolic extracts from flowers of *Inula britannica*, a plant species widely used in traditional Chinese medicine (TCM). It was confirmed that this compound was highly safe as no cytotoxicity was observed at high concentrations. Melanin production was reduced in a dose-dependent manner by this lactone (in vitro studies). A significant reduction in melanin biosynthesis was also observed in an in vivo model (zebrafish embryos). The authors discovered safe and effective natural compound for skin whitening and preventing abnormal hyperpigmentation.

In another article by Gaweł-Bęben et al. [20], comparative phytochemical and biological activity studies of hydroglycolic extracts from flowering aerial parts of *Achillea millefolium* (Am) and *Achillea biebersteinii* (Ab) collected in Kazakhstan were performed. The aim of the study was to evaluate potential cosmetic application of (Ab). Extracts from (Ab) possessed more diverse composition than (Am) and displayed significant antiradical, tyrosinase inhibitory, and sun protective properties. In contrast to (Am), (Ab) extracts were cytotoxic against the human malignant melanoma cell line and showed a lower cytotoxicity against non-cancer keratinocytes. The extracts of (Ab) could be proposed as multifunctional ingredients of cosmetics, but their safety and irritating potential should be investigated in the next step of research.

Three original articles focus on plant biotechnology methods and present the possibility to use the in vitro cultures as a potential source of bioactive natural products.

Szypuła et al. [21] proposed the in vitro cultures of gametophytes of the European club-moss species *Huperzia selago* as a rich source of two potent reversible acetylcholinesterase inhibitors: huperzine A (HA) and huperzine B (HB). They documented that the contents of (HA) were higher than in the *H. serrata* Asian species grown in open air, which is a current source of (HA) for pharmaceutical industry. The authors confirmed for the first time the presence of (HB) in in vitro cultures of *H. selago* gametophytes and identified eight new alkaloids previously unreported in this species.

Klimek-Szczykutowicz et al. [22] established that in vitro culture of *Nasturtium officinale* maintained in RITA^®^ bioreactors could be a rich potential source of glucosinolates (gluconasturtiin and 4-methoxyglucobrassicin) and rutoside, famous plant antioxidants. The biomass extracts possess the antioxidant (CUPRAC, FRAP, DPPH assays), antibacterial and antifungal activity comparable with plant herb extracts. Anti-inflammatory assays documented a higher inhibition of COX-2 for biomass cultured in vitro. The authors propose the rapidly grown biomass in bioreactors as an alternative source of bioactive compounds. 

Makowski et al. [23] present the effects of biotic (*Cronobacter sakazakii*) and abiotic (shaking—hydromechanical stress) elicitation of cells from in vitro culture of the carnivorous endemic plant species grown in North and South Carolina—*Dionaea muscipula*. The best results (increase in fresh weight, higher total phenolic content, elevated synthesis of phenolics, and plumbagin) were observed after the combined elicitation. The authors also estimated the antioxidant and antibacterial activity. A higher antioxidant activity of the extracts corresponded with a higher phenylpropanoid content. The authors concluded that only in vitro culture should be proposed for pharmaceutical purposes, because the natural populations of this plant species are very limited.

One article deals with the use of natural compounds against plant diseases. Sofy et al. [24] proposed natural compounds, glycine betaine (GB) and chitosan (CH), alone or in combination, as natural compounds, which could be an alternative to chemical pesticides in the treatment of cucumber infected with mosaic cucumovirus. The authors underlined that the combined use of (GB) and (CH) evoked the best effect against virus infection. They also highlighted that their study is the first to demonstrate the induction of systemic plant resistance against virus, by using (GB).

The next article presents a new proposal for microscopic technique with the use of semi-synthetic curcumin derivatives. Obregón-Mendoza et al. [25] synthesized mono, di-, tri-, and tetra-benzylated and dibenzylfluoroborate curcumin derivatives to obtain some ideal fluorophores. Dibenzyl (DB) and dibenzylfluoroborate (DBF) fluorophores possessed the highest fluorescence properties. Both (DB) and (DBF) made it possible to observe membranes of living cells. (DB) excited with blue or green laser allowed the observation of the nucleus. On the other hand, (DBF) was a good tool to observe the cytoplasm or exclusively the nucleus using different lasers. The authors proposed these two new fluorophores as an attractive alternative to known fluorescent dyes for the use in biomedical sciences.

Some articles draw the attention of the readers to the possibility of the use of natural products of non-plant origin.

One of them by Chang et al. [26] documented the possibility to use extraction waste of *Antrodia cinnamomea* (earlier *Ganoderma camphoratum*), a mushroom species endemic to Taiwan, in aquaculture (fish) industry. This mushroom species is produced in China in extremely high quantities as an anti-inflammatory, anti-tumor, and immunomodulatory agent. The high amounts of extraction waste rich in proteins, carbohydrates, and lipids and also containing bioactive triterpenoids led the authors to recommend it for application as a supplement in aquaculture feeds. These waste extracts inhibited the growth of microorganisms in water, increased feed efficacy in zebrafish model, improved the tolerance of zebrafish to dramatic temperature changes, enhanced its regenerative ability, and reduced symptoms of inflammation.

The article by Mahdi et al. [27] demonstrated the chemical profile of ethanolic extracts and antimicrobial activity of termites *Macrotermes bellicosus*. This termite is used in nutrition and traditional medicine in Benin for treatment of inflammatory and infectious diseases. The tested extracts were prepared from soldier and worker caste, termite mound, and fungus comb. Only the soldier caste showed antimicrobial activity against various bacterial strains. The most active compounds were identified for the first time and included hydroquinone, methylhydroquinone, 3,4-dihydroxyphenylethyl glycol, and *N*-acetyldopamine. They contributed to the growth inhibition of *S. aureus*. Additionally, the authors confirmed the presence of fatty acids in the extracts, which highlights the nutritional value of soldier caste extracts. The results provide the rationale for the traditional medicinal use of *M. bellicosus*.

Three review articles address important scientific issues.

Mączka et al. [28] collected data on potential therapeutic value of geraniol, very famous fragrance component in cosmetics with rose-like aroma. It possesses antioxidant, anti-inflammatory, and anticancer activity. Special attention was devoted to anti-cancer and antimicrobial activity of this compound. The authors underline the low toxicity and high anti-cancer potential of this compound in dangerous cancers (prostate, bowel, liver, kidney, and skin cancer).

The review by Madende and Hayes [29] is devoted to plant biostimulants as a promising alternative to conventional chemical fertilizers. The authors focus in particular on the fish protein hydrolysates, which could be produced from fish processing waste. They also present the legislation governing the use of biostimulants in agriculture and horticulture.

Ekiert et al. [30] present the position of *Artemisia vulgaris* L. in the history of medicine, its present use in traditional Chinese, Hindu, and European medicine, and current knowledge about the phytochemicals and pharmacology of this plant species. 

In summary, we are confident that the readers of *Molecules* will find this Special Issue exciting reading that will expand their scientific horizons, and that the results it presents will provide inspiration for their own research in the near future.
